# The Hospitals Association

**Published:** 1892-06-25

**Authors:** 


### THE HOSPITALS ASSOCIATION.

An influentially attended meeting of the Hospitals Association, being the eighth annual meeting, was held on Saturday evening at Charing Cross Hospital, Lord Sandhurst presiding.

The Secretary (Mr. P. Michelli) read the report, in which reference was made to the passing, through the instrumentality of the association, of the Mortmain and Charitable Uses Act, 1891, by which the obstacles that had hitherto diverted many benefactions destined for charity had been removed, and a fruitful source of vexatious litigation done away with. Members were reminded that among other practical results of their work were the Royal National Pension Fund for Nurses and the New Street Ambulance Service for London. Last year 1,000 accident cases were removed to the hospitals on the ambulance of this service. With a view to rendering it as complete as possible, a class had been formed from among the shelter attendants for instruction in first aid to the wounded, and it was hoped that the success which had attended the class would encourage others in large numbers to profit by the example. The subject of the rating of hospitals had engaged attention during the year, and efforts were being made to secure the exemption of voluntary hospitals from rating.

Lord Sandhurst in moving the adoption of the report, said he was in hearty sympathy with the work of the association, except with regard to the question of the exemption of hospitals from rating, and he was bound to say that should such a proposal come before the House of Lords, he should oppose it. He believed such a step would lead to the introduction of rate-paid officials into the hospitals, and that, he held, to be very undesirable. Having been Chairman of the House of Lords' Committee, which for three years had been enquiring into the whole subject of hospitals, and having seen nearly all the principal hospitals on the Continent, he might claim to speak with some knowledge of the matter. With the exception of one hospital in Berlin, he had seen nothing on the Continent to equal the voluntary hospitals of London. The efficiency of the hospital he had referred to in Berlin was, he believed, due to the system of nursing established by the Empress Frederick. As they were on the eve of Hospital Sunday, it was only right he should say that the Lords' Committee had come to the unanimous opinion that the system of voluntary hospitals in London was very good, and that they would be extremely sorry to see individual hospitals interfered with for the reason that the rivalry they promoted tended to their administrative efficiency. (Cheers.) The Committee were particularly struck with the able manner in which the Secretaries of the metropolitan hospitals discharged their duties.

Mr. Burdett seconded the motion. He said it was with the liveliest satisfaction that he had heard Lord Sandhurst's statement with reference to the decision of the House of Lords' Committee. After that statement, the army of hospital critics ought to cease their criticisms and give much cash. Never were the hospitals more in need of funds, so that the report of the Lords' Committee came at a very opportune moment. He was glad to hear the Secretaries so highly spoken of, for in many instances they were the backbone of the hospitals to which they were attached, and in his opinion they were not sufficiently well paid for the work they did. Referring to the street ambulance branch, he said it was an excellent piece of work, reflecting the greatest credit upon Mr. Thomas Ryan, the Hon. Secretary, who had given up nearly the whole of his leisure to it, and had succeeded in making the organisation complete and prosperous. Mr. H. L. Bischoffsheim, with a liberality worthy of the widest publicity, had undertaken to defray, out of his own pocket, the whole cost of the street ambulance, both for maintenance and material. He would never have taken this step had Mr. Ryan failed to convince him that the organisation was one of essential importance to the citizens of London. Such quiet munificence as Mr. Bischoffsheim had exhibited was worthy of wide imitation, and would, he was certain, be gratefully recognised by the members of the Association.

The motion was agreed to, and a vote of thanks having been passed to Lord Sandhurst for presiding, the proceedings closed.

